# Optimization of cassava peel ash concrete using central composite design method

**DOI:** 10.1038/s41598-024-58555-0

**Published:** 2024-04-04

**Authors:** Uzoma Ibe Iro, George Uwadiegwu Alaneme, Imoh Christopher Attah, Nakkeeran Ganasen, Stellamaris Chinenye Duru, Bamidele Charles Olaiya

**Affiliations:** 1 Department of Civil Engineering, Michael Okpara University of Agriculture, Umudike, Nigeria; 2 Department of Civil, School of Engineering and Applied Sciences, Kampala International University, Kampala, Uganda; 3 Department of Civil Engineering, Akwa Ibom State University, Ikot Akpaden, Nigeria; 4 Department of Civil Engineering, SRM Institute of Science and Technology, Kattankulathur, Chengalpattu, 603203 Tamil Nadu India; 5 Agricultural and Bioresources Engineering Department, University of Nigeria, Nsukka, Nigeria

**Keywords:** Response surface methodology, Analysis of variance, Agro-waste, Design expert, Engineering, Materials science

## Abstract

Cassava peel ash (CPA) is an abundant agricultural byproduct that has shown promise as an additional cementitious material in concrete manufacturing. This research study aims to optimize the incorporation of CPA in concrete blends using the central composite design (CCD) methodology to determine the most effective combination of ingredients for maximizing concrete performance. The investigation involves a physicochemical analysis of CPA to assess its pozzolanic characteristics. Laboratory experiments are then conducted to assess the compressive and flexural strengths of concrete mixtures formulated with varying proportions of CPA, cement, and aggregates. The results show that a mix ratio of 0.2:0.0875:0.3625:0.4625 for cement, CPA, fine, and coarse aggregates, respectively, yields a maximum compressive strength of 28.51 MPa. Additionally, a maximum flexural strength of 10.36 MPa is achieved with a mix ratio of 0.2:0.0875:0.3625:0.525. The experimental data were used to develop quadratic predictive models, followed by statistical analyses. The culmination of the research resulted in the identification of an optimal concrete blend that significantly enhances both compressive and flexural strength. To ensure the reliability of the model, rigorous validation was conducted using student’s t-test, revealing a strong correlation between laboratory findings and simulated values, with computed p-values of 0.9987 and 0.9912 for compressive and flexural strength responses, respectively. This study underscores the potential for enhancing concrete properties and reducing waste through the effective utilization of CPA in the construction sector.

## Introduction

Concrete stands as one of the extensively utilized construction materials worldwide; however, its manufacturing contributes considerably to environmental consequences because of the substantial energy consumption and carbon emissions linked to cement production^1,2^. In response to these environmental considerations and to advocate for sustainable construction methods, researchers are progressively investigating alternative materials and mix formulations. One such material of interest is cassava peel ash (CPA), a waste product generated from cassava processing^3^. Cassava (Manihot esculenta) is a vital crop in many tropical countries, and its processing generates substantial amounts of waste, primarily in the form of cassava peels. Improper disposal of cassava peels can lead to environmental pollution and health hazards ^4^. However, recent research has shown that these waste cassava peels can be effectively converted into ash, known as cassava peel ash (CPA), and utilized as a supplementary material in concrete production. CPA contains pozzolanic properties, similar to other supplementary cementitious materials like fly ash or silica fume. Pozzolanic substances have the capability to interact with calcium hydroxide in cement, resulting in the formation of supplementary cementitious compounds, ultimately enhancing the properties of concrete^4,5^. However, the optimization of CPA's incorporation into concrete mixes is essential to ensure the desired performance characteristics are achieved^6^.

The incorporation of CPA in concrete has gained attention due to its pozzolanic characteristics, which can contribute to enhanced strength, durability, and reduced environmental impact. The pozzolanic nature of cassava peel ash indicates its capacity to undergo a reaction with calcium hydroxide in the presence of moisture, resulting in the formation of extra cementitious compounds^7^. This chemical process contributes to the reinforcement of strength and improvement of the durability of concrete. Moreover, CPA have found a wide acceptance in their utilization in civil engineering materials like concrete and soil re-engineering. In recent times utilization of this agro waste derivatives as supplementary cementitious material has been practiced to enhance concrete’s mechanical properties when used in cement replacement strategy^8,9^. Several research investigations have explored the possibilities of incorporating CPA in concrete applications. Ogunbode et al.^10^ explores the mechanical and microstructure properties of concrete composites made using CPA and kenaf bio-fibers. The study likely investigates the potential for incorporating these sustainable materials into concrete mixtures. Also, in research carried out by Olubunmi et al.^11^, they investigate the use of cassava peel ash and wood ash as partial cement replacements in concrete. Various replacement percentages were tested, with 5%, 10%, and 15% replacements meeting plain concrete strength specifications. Higher percentages, such as 20% and 25%, were unsuitable for structural concrete. The study suggests that incorporating these materials into concrete production can help reduce environmental pollution.

Optimization of cassava peel ash (CPA) concrete using the Central Composite Design (CCD) method is an innovative approach that aims to improve the properties and performance of concrete by incorporating cassava peel ash as a supplementary cementitious material^12,13^. Statistical analysis of the experimental data enables researchers to model the relationship between the variables and the response using regression techniques and response surface methodology^14^. This allows for the identification of significant factors, evaluation of their individual and interactive effects, and determination of the optimal parameter values that maximize the desired response^15^. The optimization of CPA concrete using the CCD method offers several advantages, including reduced time and cost compared to traditional trial-and-error approaches. It enables researchers to efficiently explore a wide range of parameters and their interactions, leading to improved understanding and control over the properties of CPA concrete^16,17^. By identifying the optimal combination of variables, it is possible to enhance the performance, sustainability, and economic viability of concrete structures. It also provides a systematic and data-driven approach to guide the selection and proportioning of materials, ultimately leading to improved concrete performance and sustainability^18^.

Lately, many researchers have employed unconventional techniques to assess concrete performance concerning the interplay of mix ingredients^19–21^. These methods encompass statistical, computational, and analytical approaches. Hassan et al.^22^ evaluate the use of micro and nano palm oil fuel ash (POFA) as supplementary cementitious materials in high-strength blended concrete. The research aims to optimize the concrete mix proportions using Central Composite Design and Response Surface Methodology. The experimental results validate mathematical models, indicating a close agreement between predictions and data. The study suggests an optimal mix with 10% micro POFA and 1.50–2.85% nano POFA, meeting optimization criteria for fresh and hardened concrete properties. Moreover, Ali et al.^23^ investigate the use of pumice stone (PS) as a replacement for natural coarse aggregates in concrete. Various percentages of PS are used in the mix, and response surface methodology (RSM) is employed for experimentation. The study suggests that up to 30% of PS can be replaced in lightweight aggregate concrete, resulting in compressive strength greater than 15 MPa, split tensile strength at 7–12% of CS, and flexural strength at 9–11% of CS. The proposed quadratic model is highly relevant, with a coefficient of determination (R^2^) above 99% for all responses. Also, Ali et al*.*^24^ researched on the utilization of waste foundry sand (WFS) as a partial replacement for fine aggregate in concrete mixtures and assess its impact on fresh concrete performance and mechanical properties. WFS ratios were adjusted using Design-Expert software's Central Composite Design (CCD) tool in Response Surface Methodology (RSM). Results showed highest mechanical properties at 20% WFS replacement and 56 days curing, with compressive strength of 29.37 MPa, splitting tensile strength of 3.828 MPa, and flexural strength of 8.0 MPa. However, upto 30% replacement, fresh qualities of substitutes were akin to the control mix.

Furthermore, the optimization of cassava peel ash concrete using the Central Composite Design method is a valuable research approach that allows for the systematic exploration and optimization of various variables to enhance the properties and performance of concrete and provides a systematic and data-driven approach to improve the properties and performance of concrete^13,25^. Utilizing this method, researchers can maximize the utilization of cassava peel ash, a waste material, while improving the performance, sustainability of concrete structures and contribute to the development of more resource-efficient construction materials^26^. The study aims to optimize parameters for concrete with CPA utilizing the CCD method. This approach facilitates a systematic exploration of various variables and their interactions to identify the optimal combination that achieves the desired properties in the concrete. By employing statistical analysis and response surface modeling, the study aims to develop a comprehensive understanding of the relationship between the variables and the response, enabling the identification of the optimal parameter values.

The outcomes of this research will provide valuable insights into the optimization of CPA concrete, enabling more efficient utilization of cassava peel ash and enhancing the sustainability and performance of concrete structures. Ultimately, this study is motivated by several factors. Firstly, it aims to promote sustainable and eco-efficient construction practices by utilizing agricultural waste in concrete production. Secondly, there is potential for economic benefits through cost savings by replacing traditional cement with CPA. Additionally, the study seeks to enhance concrete performance by systematically exploring different mixture formulations using advanced design methodologies. The utilization of technology like Design Expert software streamlines the optimization process and contributes to advancements in sustainable construction practices. Overall, the research aims to improve concrete sustainability, cost-effectiveness, and performance through the effective integration of CPA.

## Materials and methods

### Materials

#### Cement

The experimental investigation utilized Grade 53 Dangote cement, obtained from the open market for building materials in Imo State, Nigeria. Furthermore, it adheres to the standards, composition, and compliance requirements outlined in BS 12 (1978).

#### Water

Water plays a crucial role as a component in the concrete mixture, influencing the mechanical, rheological, and durability properties. For the laboratory tests, we employed potable water that complies with the specifications outlined in ASTM C1602-12 (2012) for concrete applications.

#### Aggregates

In this experimental study, we employed river sand sourced from Akwa Ibom State, Nigeria, as the fine aggregate. The fine aggregate employed meets the criteria outlined in BS-EN 12,620 and ASTM C125-16 and can pass through a 2.36 mm sieve. As for the coarse aggregate, crushed granite with well-graded properties and devoid of harmful substances was employed, and adherence to BS EN12620. The coarse aggregate has a maximum size of 20 mm.

#### Cassava peel ash (CPA)

**Figure 1:**
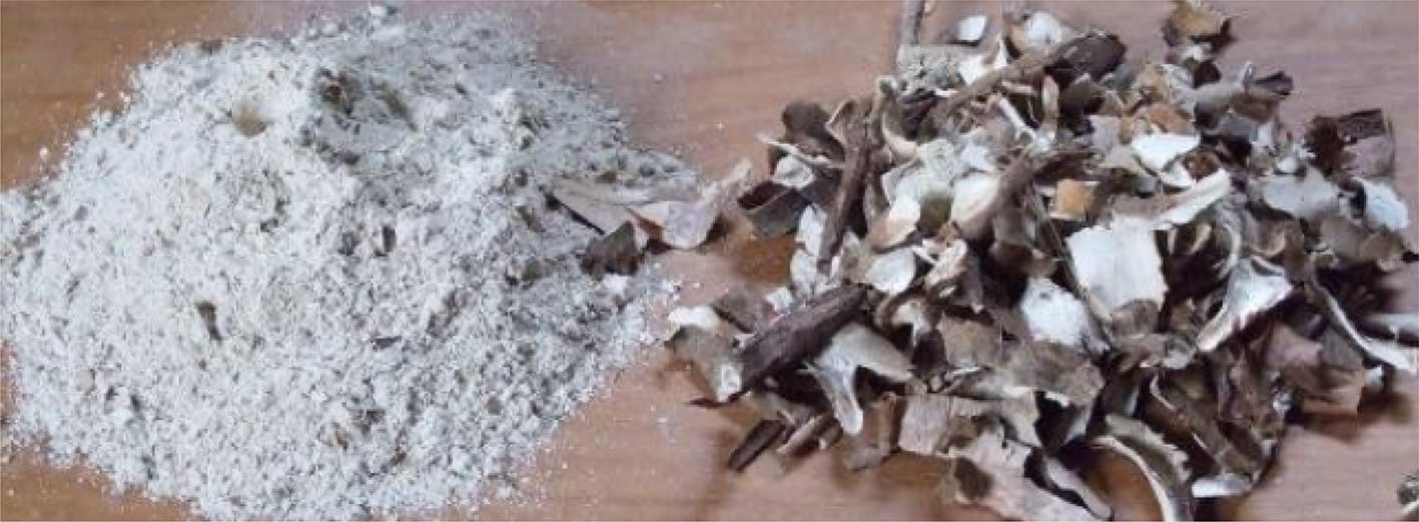
Ash samples derived from cassava peel.

The cassava peel was collected from Abayi-umuokoroato village, situated in the Abayi Ancient Kingdom of Obingwa Local Government Area in Abia State, Nigeria. Subsequently, the cassava peel was subjected to sun drying. It was then incinerated in a controlled kiln at a temperature range of approximately 500 °C to 850 °C for 60 min to ensure environmental protection. The resulting burnt material was carefully gathered and sieved in the laboratory, using a 150 µm sieve size, to obtain finely divided ash material for the experiments. The picture of the cassava peel waste taken in the laboratory during the experiments along with the processed ash samples are shown in Fig. 1

### Design of experiment using CCD

Response Surface Methodology is a statistical method employed for experiment design to uncover relationships between variables and responses. Its primary goal is optimizing these variables to anticipate the most favorable responses^27^. CCD is a valuable technique for establishing a functional connection between the variables and responses. it incorporates a nested factorial or fractional factorial design with central points, enhanced by a set of 'star points' for curvature estimation. While the center-to-factorial point distance is ± 1 unit for each factor, the center-to-star point distance is |α|> 1. The specific value of α is determined based on design requirements and the number of factors in question, however, Face Centered Central Composite Design (FCCD) which have all the axial points are projected on the surfaces was utilized for the formulation^28^. Design Expert 13.0.5.0 Software was used for designing the experiments, mathematically modeling, statistically analyzing, and optimizing the response parameters. In essence, the Central Composite Design (CCD) includes 2n factorial experiments along with 2n axial experiments, and the experimental error is assessed using center point replicates (n_c_). Therefore, a Face Centered Central Composite Design (FCCD) comprises 2n factorial runs, coded as + −1, expanded by 2n axial points like (1, 0, 0…0), (0, + −a, 0…0), …(0, 0, + −… 0), and n_c_ center points (0, 0, 0...0). The total number of required experimental runs (N) for CCD is determined by Eq. (1)^29^.

1 $$ {\text{N}}\, = \,{2}^{{\text{n}}} \, + \,{\text{n}}_{{\text{c}}} \, + \,{\text{2n}}\, = \,{2}^{{4}} \, + \,{1}\, + \,{2}\, \times \,{4}\, = \,{25} $$

**Figure 2:**
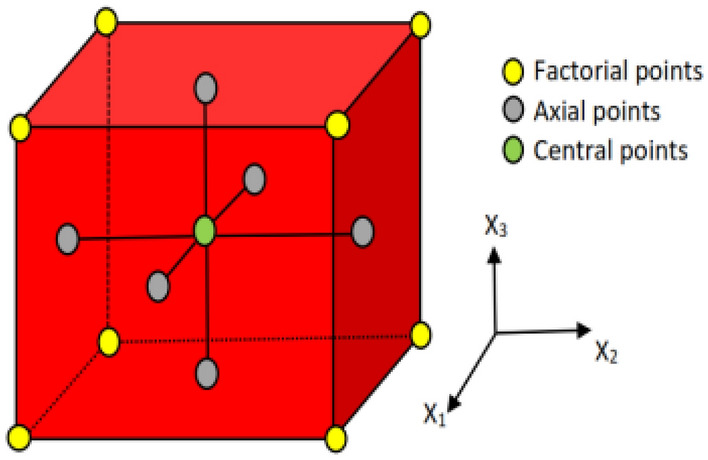
FCCD diagrammatic illustration^29^.

In this context, n represents the number of variables, while n_c_ pertains to the number of central points. For our study, which incorporated four input variables, we adopted a CCD design consisting of twelve factorial points, eight axial points, and a single repetition at the center. The arrangement of these points can be visualized in Fig. 2. Consequently, we conducted a total of twenty-five experimental runs, considering four parameters, each varying at three levels denoted as − 1, 0, and 1^30^.

#### Formulation of mixture components ratio

**Figure 3:**
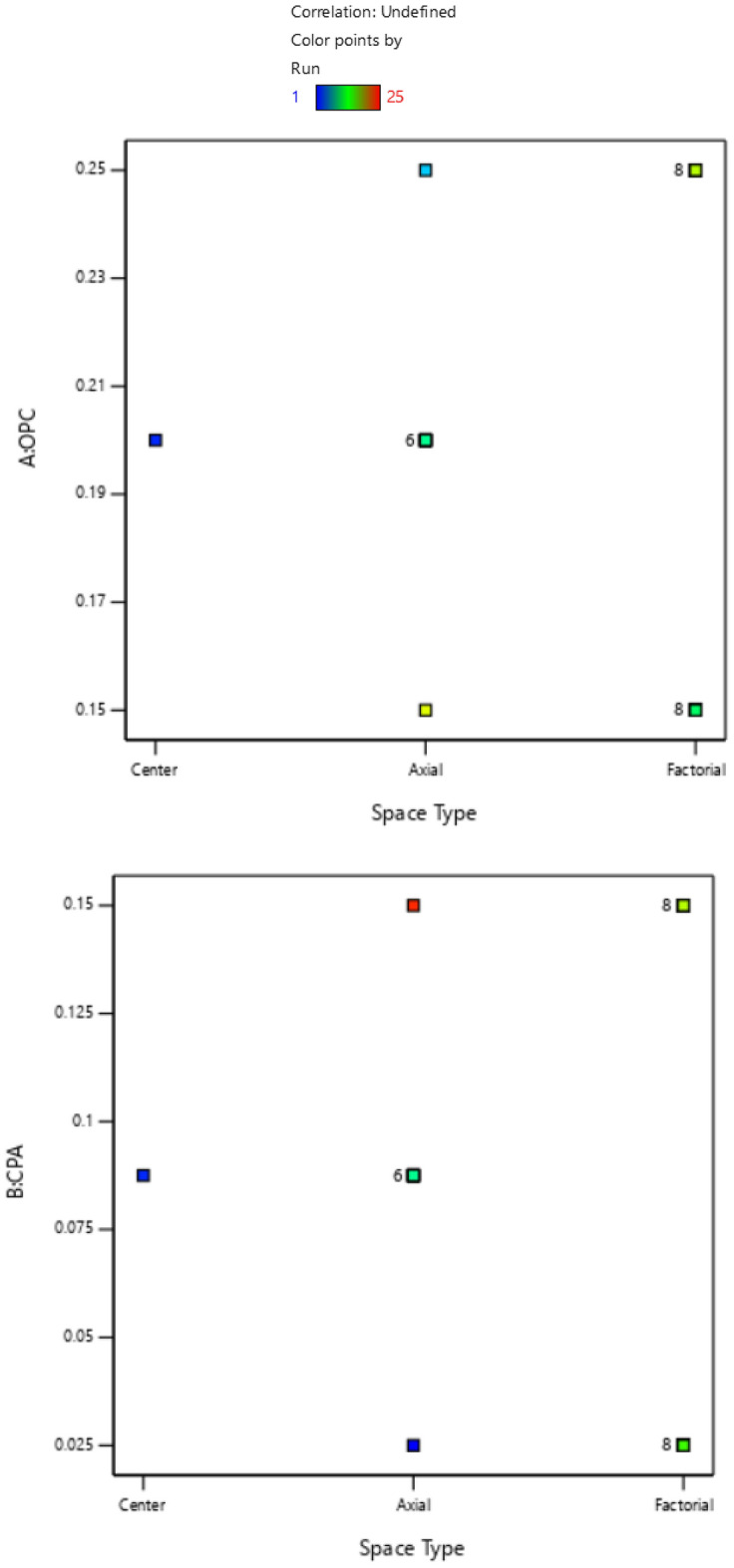

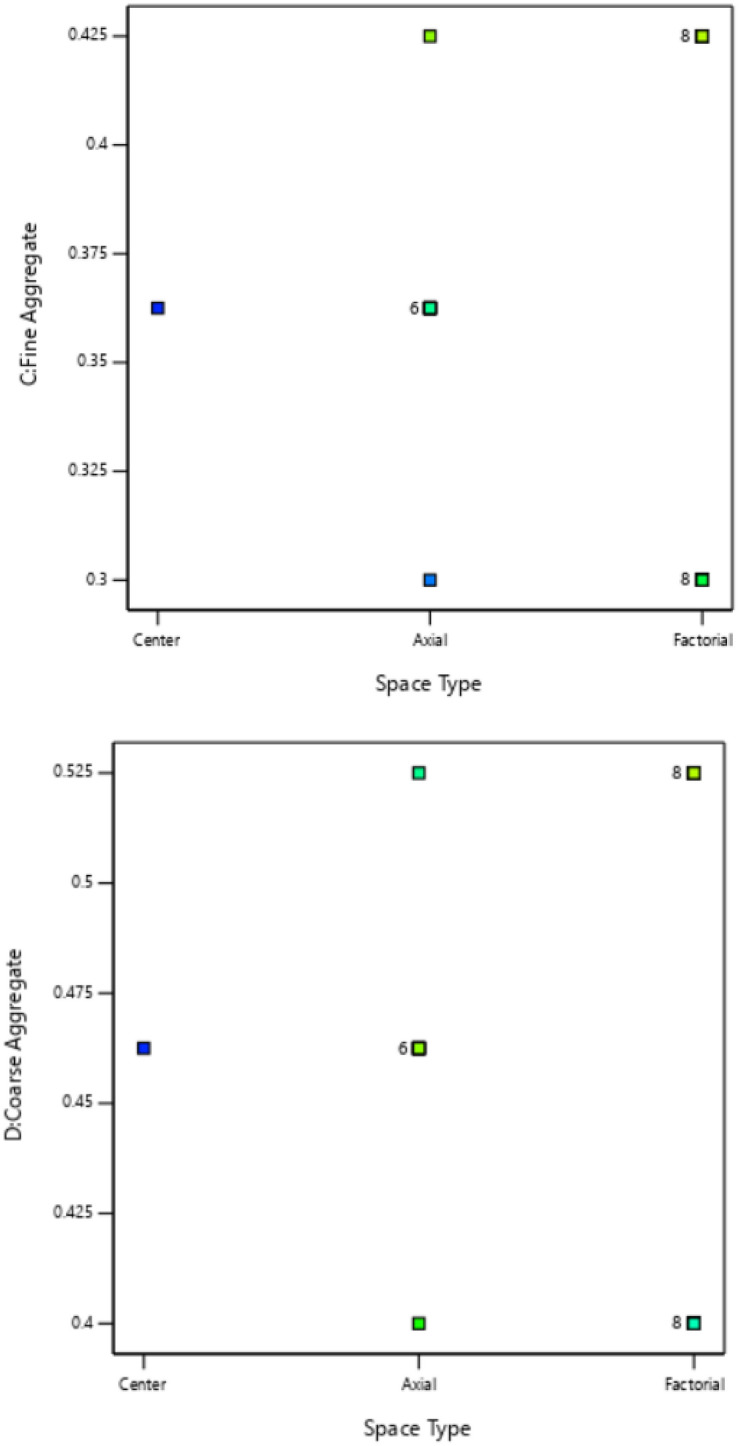
Experimental factor space.

**Figure 4:**
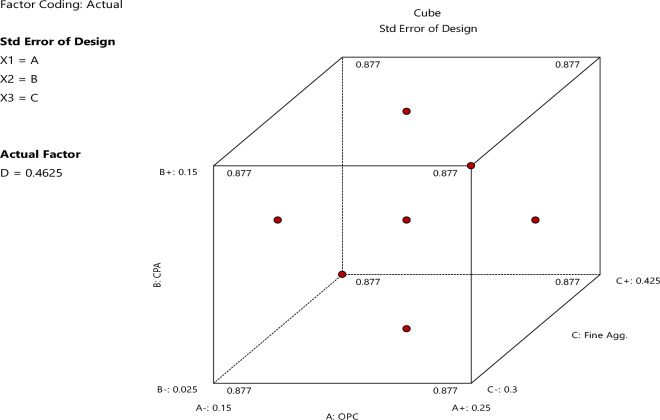
Cube standard error of design.

In CCD, mix design refers to the process of determining the composition of the experimental mixtures that will be used in the study. The methodological approach involves selecting appropriate levels or values for the variables being studied and preparing the experimental mixtures accordingly^31^. The mix design process in central composite design involves carefully selecting variable ranges, determining design points, assigning variable levels, calculating ingredient proportions, and preparing the experimental mixtures. This allows for a systematic exploration of the variable space and helps in understanding the interactions between the factor levels and the target response(s) of interest. The collected data from the experiments can then be used for statistical analysis and optimization to ascertain the optimal mix composition that achieves the desired objectives of the study^32,33^. The concrete mix design parameters for this experimental study indicated target strength of 25 N/mm^2^, with a cement content of 290 kg/m^3^, coarse aggregate content of 1198.65 kg/m^3^, and fine aggregate content of 766.35 kg/m^3^ which were derived from relevant literature^34,35^. Furthermore, taking water-cement-ratio (w/c) of 0.5, the central composite design mixture formulation obtained with the aid of design expert software for the experimental investigations showing four components’ constituents of cement, cassava peel ash (CPA), fine and coarse aggregates is shown in Tables 1,2. Moreover, the experimental factor space for the four components in the mixture design and the cubic plot standard error of design were presented in Figs. 3,4. The plot displayed the factor space on the x-axis, illustrating three sections (center, factorial, and axial) for the central composite design using Design Expert Software. Meanwhile, the mixture components' ratios for the 25 experimental runs were depicted on the y-axis of the plot. Additionally, it was noted that 16 out of the 25 design points are located on the factorial plane within the factor space. Among these, eight data points are positioned at both the lower and upper limits for the four mixture components^36,37^.

**Table 1:** Mixture factors build information.

Factors	Minimum	Maximum	Coded low	Coded high	Mean	Std. dev
OPC	0.15	0.25	− 1 ↔ 0.15	+ 1 ↔ 0.25	0.2000	0.0433
CPA	0.025	0.15	− 1 ↔ 0.02	+ 1 ↔ 0.15	0.0875	0.0541
Fine aggregate	0.3	0.425	− 1 ↔ 0.30	+ 1 ↔ 0.42	0.3625	0.0541
Coarse aggregate	0.4	0.525	− 1 ↔ 0.40	+ 1 ↔ 0.53	0.4625	0.0541

**Table 2:** Experimental mixture formulation.

	Actual units					Coded units			
S/n	OPC	CPA	Fine aggregate	Coarse aggregate	Response	OPC	CPA	Fine agg	Coarse agg
1	0.2	0.025	0.3625	0.4625		0	− 1	0	0
2	0.2	0.0875	0.3625	0.4625		0	0	0	0
3	0.15	0.15	0.3	0.525		− 1	1	− 1	1
4	0.2	0.0875	0.3	0.4625		0	0	− 1	0
5	0.15	0.025	0.425	0.4		− 1	− 1	1	− 1
6	0.25	0.0875	0.3625	0.4625		1	0	0	0
7	0.15	0.025	0.3	0.525		− 1	− 1	− 1	1
8	0.15	0.15	0.425	0.4		− 1	1	1	− 1
9	0.25	0.15	0.425	0.4		1	1	1	− 1
10	0.2	0.0875	0.3625	0.525		0	0	0	1
11	0.15	0.15	0.425	0.525		− 1	1	1	1
12	0.25	0.15	0.3	0.525		1	1	− 1	1
13	0.25	0.025	0.425	0.4		1	− 1	1	− 1
14	0.2	0.0875	0.3625	0.4		0	0	0	− 1
15	0.25	0.025	0.425	0.525		1	− 1	1	1
16	0.15	0.025	0.425	0.525		− 1	− 1	1	1
17	0.2	0.0875	0.425	0.4625		0	0	1	0
18	0.25	0.15	0.425	0.525		1	1	1	1
19	0.15	0.0875	0.3625	0.4625		− 1	0	0	0
20	0.15	0.025	0.3	0.4		− 1	− 1	-1	− 1
21	0.25	0.025	0.3	0.525		1	− 1	− 1	1
22	0.25	0.025	0.3	0.4		1	− 1	− 1	− 1
23	0.25	0.15	0.3	0.4		1	1	− 1	− 1
24	0.15	0.15	0.3	0.4		− 1	1	− 1	− 1
25	0.2	0.15	0.3625	0.4625		0	1	0	0

### Compressive strength property

The mixture components were accurately weighed and thoroughly mixed based on the specified formula. The resulting uniform concrete mixture was compacted into 150 mm × 150 mm × 150 mm cubic molds. These green concrete specimens, blended with CPA, were submerged in a curing tank filled with clean water for 28 days at normal temperature. After the curing period, they were weighed, and their compressive strength was determined following the BS EN 12,390-4 standard. The cubes underwent crushing tests using the Okhard Machine Tool’s WA-1000B digital display Universal Testing Machine, with a testing range of 0–1000kN. The cubes were positioned between two 25 mm-thick steel plates that covered the top and bottom, and force was incrementally applied until the cubes failed in compression^38,39^.

### Flexural strength

The procedure for the flexural strength test will adhere to BS EN 12,390-5 (2009) standards, utilizing test specimens with dimensions of 400 × 100 × 100 mm. These specimens will be thoroughly batched and mixed in accordance with the specified component fractions. Subsequently, the concrete beams formed will be demolded and allowed to cure for a 28-day hydration period before undergoing the flexural test. After twenty-eight days of curing, three samples from each experimental run will be subjected to testing, and the average flexural strength will be determined. This process will be repeated for each mix proportion, testing three specimens per proportion and calculating the average flexural strength for each^40^.

### Ethics and compliance statement

Authors comply with the International Union for Conservation of Nature (IUCN) Policy Statement on Research Involving Species at Risk of Extinction and the Convention on the Trade in Endangered Species of Wild Fauna and Flora in this research article.

### Consent to participate

All authors were highly cooperative and involved in research activities and preparation of this article.

## Results discussion and analysis

### Test materials characterization

**Figure 5:**
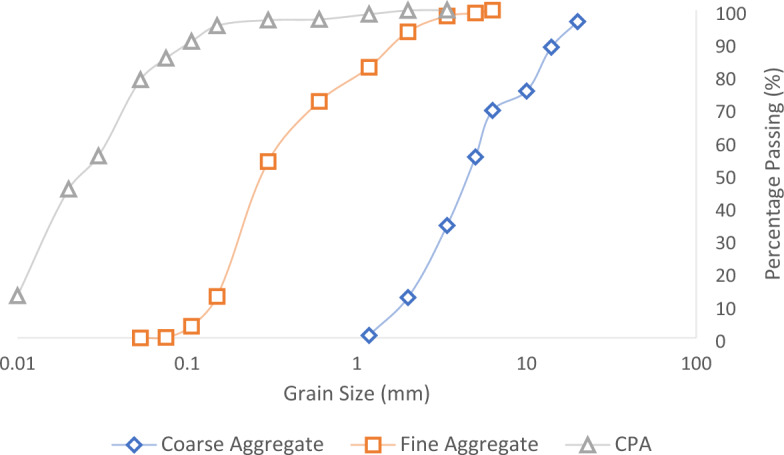
Particles size distribution of test ingredient.

A sequence of laboratory examinations was carried out on the constituent elements to evaluate their suitability as construction materials in civil engineering. The examinations encompassed sieve analysis and specific gravity tests on aggregates and admixtures to assess particle size distribution and gradation. The results of the sieve analysis test are presented in Fig. 5, depicting the particle size variation with a cumulative frequency distribution curve. The findings revealed that the coarse aggregate exhibited a passing sieve size of 76.2–11.6% for 10–2 mm, while the fine aggregates demonstrated a passing sieve size of 93.4–0.13 for 2 mm–75 µm^41^. Moreover, the CPA admixtures in the concrete showed a passing sieve size of 99.99–84.63% for 2 mm–75 µm. The results conform to the requirements outlined by BS 882, indicating well-graded sand and gravel particles for enhanced concrete durability performance^38,39^.

#### Chemical characterization of the test cement and CPA

The chemical attributes of the examined admixtures were assessed through X-ray fluorescence (XRF). The results revealed that CPA consists of Fe_2_O_3_ (6.02%), Al_2_O_3_ (19.88%), and SiO_2_ (55.93%), and totaling 81.83% composition, indicating a favorable pozzolanic property compliant with ASTM C618, 98 specifications^40^. Furthermore, the cement composition indicated 9.85% for CaO, 51.4% for SiO_2_, and 20.6% for Al_2_O_3_. The plentiful presence of these elemental oxides in the examined materials supports extensive cement hydration, improving the mechanical strength and longevity of the resulting environmentally friendly concrete, as depicted in Table 3. The reaction mechanism of hydration enables the amalgamation of aluminate and silicate oxides from the admixture with hydrated calcium, leading to the formation of a more robust mass over time^41^.

**Table 3:** Chemical properties of test cement (OPC) and CPA.

Oxides	MgO	Na_2_O	CaO	Fe_2_O_3_	MnO	K_2_O	SiO_2_	TiO_2_	Al_2_O_3_	LOI	ZnO
OPC	0.093	1.05	13.45	6.53	Trace	1.6	51.4	0.52	20.6	3.9	Trace
CPA	3.2	0.98	9.85	6.02	1.55	Trace	55.93	Trace	19.88	12.5	1.4

### Effects of CPA admixtures on the mechanical laboratory response

**Figure 6:**
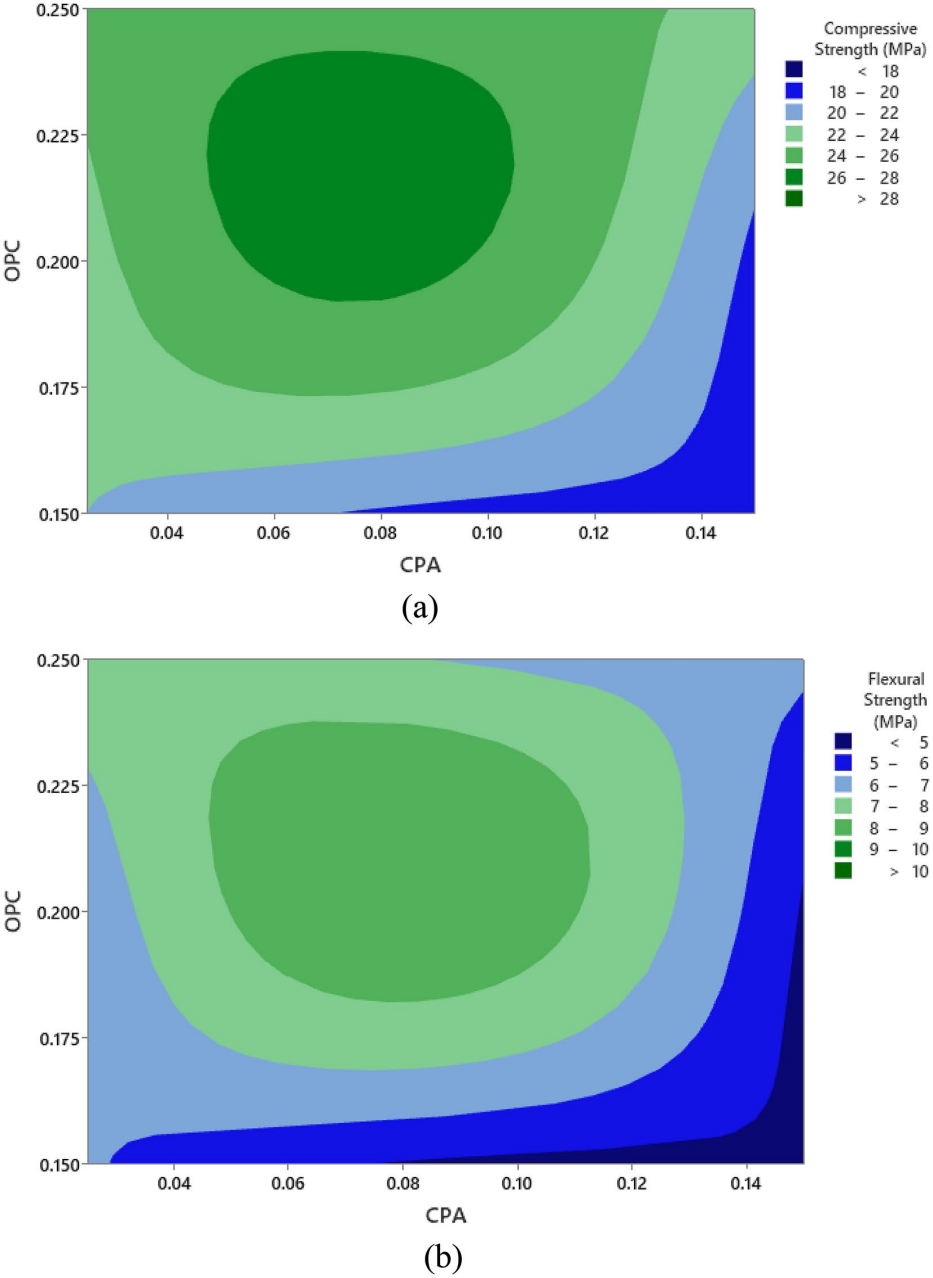
Impact of the interaction between CPA and cement on (a) Compressive Strength and (b) Flexural Strength.

The effective mass values for the ingredients were determined using the ratio conversion method, ensuring precise measurements for each experimental run with a w/c of 0.5. This conversion took into account the standard concrete density of 2400 kg/m^3^ and applied the relationship between volume, density, and mass^42^. The mass required to fill the cubic mold was determined by multiplying the calculated mold volume (m^3^) by the concrete density. For each experimental run, three cube and beam samples were produced, and the average compressive strength response is provided in Tables 4–5. The graphical representation of the influence of cement and CPA interactions on the compressive and flexural strength responses is presented in Fig. 6. The contour plot illustrates a consistent rise in both compressive and flexural strength attributes of the CPA-blended concrete as the proportion of CPA replacing cement in the mixture gradually increases from 0.025 to 0.875. However, the strength responses begin to decline with further increments in the CPA ratio, particularly at 0.12 and beyond. The maximum compressive strength recorded was 28.51 MPa, achieved with a concrete mixture ratio of 0.2:0.0875:0.3625:0.4625 for cement, CPA, fine, and coarse aggregates. Conversely, the minimum compressive strength of 17.25 MPa corresponded to a mixture ratio of 0.15:0.15:0.425:0.525. Moreover, incorporating 19% cement, 2.4% CPA, 34.6% fine aggregate, and 44% coarse aggregates notably enhanced the compressive strength behavior of the green concrete. Additionally, the highest flexural strength of 10.36 MPa was achieved with a mixture ratio of 0.2:0.0875:0.3625:0.525, and the lowest flexural strength of 4.22 MPa was observed with a mixture ratio of 0.15:0.15:0.425:0.525. Furthermore, obtained results showed that proportions of 17.02% of cement, 7.45% of CPA, 30.85% of fine aggregate and 44.68 of coarse aggregate produced best performance in terms of flexural strength of the CPA-concrete^43,44^. Overall, the concrete's mechanical strength behavior complied with NCP-1 and BS-8110 specifications, attributed to the pozzolanic properties derived from the abundance of aluminosilicate oxides in the CPA combined with Portland cement, resulting in the formation of calcium silicate hydrate^45,46^.

**Table 4:** Compressive strength results.

S/n	OPC (kg)	CPA (kg)	Fine aggregate (kg)	Coarse aggregate (kg)	Compressive strength (MPa)
1	4.63	0.58	8.39	10.70	23.29
2	4.37	1.91	7.92	10.10	28.51
3	3.24	3.24	6.48	11.34	20.68
4	4.63	2.03	6.94	10.70	27.05
5	3.65	0.61	10.33	9.72	20.09
6	5.23	1.83	7.58	9.67	25.13
7	3.65	0.61	7.29	12.76	20.22
8	3.24	3.24	9.18	8.64	18.94
9	4.96	2.98	8.43	7.93	19.12
10	4.14	1.81	7.50	10.86	26.56
11	2.92	2.92	8.26	10.21	17.25
12	4.96	2.98	5.95	10.41	23.88
13	5.52	0.55	9.39	8.84	21.76
14	4.63	2.03	8.39	9.26	26.84
15	4.96	0.50	8.43	10.41	25.67
16	3.24	0.54	9.18	11.34	21.24
17	4.14	1.81	8.79	9.56	22.82
18	4.50	2.70	7.65	9.45	23.18
19	3.43	2.00	8.29	10.58	19.57
20	4.17	0.69	8.33	11.11	26.43
21	5.52	0.55	6.63	11.60	25.92
22	6.23	0.62	7.48	9.97	26.01
23	5.52	3.31	6.63	8.84	27.14
24	3.65	3.65	7.29	9.72	20.86
25	4.14	3.10	7.50	9.56	19.47

**Table 5:** Flexural strength results.

S/n	OPC (kg)	CPA (kg)	Fine aggregate (kg)	Coarse aggregate (kg)	Flexural strength (MPa)
1	5.49	0.69	9.94	12.69	6.32
2	5.18	2.27	9.38	11.97	9.28
3	3.84	3.84	7.68	13.44	5.39
4	5.49	2.40	8.23	12.69	8.53
5	4.32	0.72	12.24	11.52	5.06
6	6.19	2.17	8.98	11.46	6.97
7	4.32	0.72	8.64	15.12	5.11
8	3.84	3.84	10.88	10.24	4.48
9	5.88	3.53	9.99	9.40	4.75
10	4.90	2.14	8.89	12.87	10.36
11	3.46	3.46	9.79	12.10	4.22
12	5.88	3.53	7.05	12.34	6.58
13	6.55	0.65	11.13	10.47	5.84
14	5.49	2.40	9.94	10.97	9.13
15	5.88	0.59	9.99	12.34	7.31
16	3.84	0.64	10.88	13.44	5.88
17	4.90	2.14	10.42	11.34	6.16
18	5.33	3.20	9.07	11.20	6.27
19	4.07	2.37	9.83	12.54	4.83
20	4.94	0.82	9.87	13.17	8.41
21	6.55	0.65	7.85	13.75	8.92
22	7.38	0.74	8.86	11.82	9.01
23	6.55	3.93	7.85	10.47	7.36
24	4.32	4.32	8.64	11.52	5.45
25	4.90	3.68	8.89	11.34	4.88

## Development and validation of the model

The information obtained from the experimental procedure, which involved the application of the prescribed proportions of mixture ingredients and the corresponding responses to assess the mechanical performance of the created CPA-cement blended concrete, was utilized for constructing the model through response surface methodology. The process involves expertly choosing square root transformation with polynomial analysis type for the purpose of considering non-linearity of the datasets to generate accurate model predictions^47^. Further statistical computations were conducted on the datasets to assess their appropriateness for the intended modeling purposes, including fit statistics and analysis of variance (ANOVA). This crucial preliminary statistical analysis provides a fit summary to identify models using performance indicators such as the coefficient of determination (Rsqd.), PRESS (predicted residual sum of squares), which evaluates how well the sought-after models fit each point in the design, lack of fit tests, and sequential model sum of squares to determine the highest polynomial order with significant additional terms, as detailed in Tables 6, 7, 8, 9. The presented fit statistical outcomes indicate a preference for quadratic models, with R-sqd. values of 0.8675 and 0.9102 for compressive and flexural strength responses, respectively. From the sequential sum of squares computation results, p value of 0.0237 and 0.0014 for compressive and flexural strength responses respectively^38,48^.

**Table 6:** Model summary statistics for compressive strength response.

Source	Std. Dev	R^2^	Adjd. R^2^	Pred. R^2^	PRESS	
Linear	0.2606	0.5086	0.4103	0.2815	1.99	
2FI	0.2716	0.6264	0.3595	− 0.0652	2.94	
Quadratic	**0.1914**	**0.8675**	**0.6821**	**0.2491**	**2.08**	**Suggested**

Significant values are bold.

**Table 7:** Sequential model sum of squares (type I) for compressive strength response.

Source	SS	df	MS	F-value	p-value	
Mean vs Total	574.87	1	574.87			
Linear vs Mean	1.41	4	0.3515	5.18	0.0050	
2FI vs Linear	0.3256	6	0.0543	0.7356	0.6295	
Quadratic vs 2FI	**0.6666**	**4**	**0.1667**	**4.55**	**0.0237**	**Suggested**
Residual	0.2468	2	0.1234			
Total	577.63	25	23.11			

Significant values are in bold.

**Table 8:** Model summary statistics for Flexural strength response.

Source	Std. Dev	R^2^	Adjd. R^2^	Pred. R^2^	PRESS	
Linear	0.2744	0.4530	0.3436	0.2430	2.08	
2FI	0.3012	0.5386	0.2090	− 0.0454	2.88	
Quadratic	**0.1572**	**0.9102**	**0.7844**	**0.4965**	**1.39**	**Suggested**

Significant values are in bold.

**Table 9:** Sequential Model Sum of Squares (SS) (type I) for Flexural strength response.

Source	SS	df	MS	F-value	p-value	
Mean vs Total	163.75	1	163.75			
Linear vs Mean	1.25	4	0.3117	4.14	0.0133	
2FI vs Linear	0.2356	6	0.0393	0.4329	0.8450	
Quadratic vs 2FI	**1.02**	**4**	**0.2557**	**10.34**	**0.0014**	**Suggested**
Residual	0.1610	2	0.0805			
Total	166.50	25	6.66			

Significant values are in bold.

### Analysis of variance (ANOVA) result

Following the identification of a suitable polynomial model, as suggested during the fit statistical analysis, ANOVA is conducted. In this step, descriptive and statistical tests are carried out to assess the significance levels of the mixture model independent variables concerning the response parameters^49^. The computational outcomes are detailed in Table 10 for the compressive strength response, indicating a Model F-value of 4.68, signifying the significance of the model. There is only a 0.94% (p-value of 0.0094) probability that an F-value of this magnitude could occur due to random variations. Additionally, the statistical results for the flexural strength response show a Model F-value of 7.24, suggesting the significance of the model as shown in Table 11. There is only a 0.17% (p-value of 0.0017) chance that an F-value of this magnitude could occur due to random variations^50^.

**Table 10:** ANOVA Quadratic model for compressive strength response.

Source	SS	df	MS	F-value	p-value	
Model	2.40	14	0.1713	4.68	0.0094	Significant
A-OPC	0.6599	1	0.6599	18.02	0.0017	
B-CPA	0.2594	1	0.2594	7.08	0.0238	
C-Fine Agg	0.4832	1	0.4832	13.20	0.0046	
D-Coarse Agg	0.0035	1	0.0035	0.0959	0.7632	
AB	0.0138	1	0.0138	0.3762	0.5533	
AC	0.0028	1	0.0028	0.0772	0.7868	
AD	0.0974	1	0.0974	2.66	0.1340	
BC	0.0164	1	0.0164	0.4473	0.5187	
BD	0.0000	1	0.0000	0.0011	0.9741	
CD	0.1952	1	0.1952	5.33	0.0436	
A^2^	0.1308	1	0.1308	3.57	0.0881	
B^2^	0.2703	1	0.2703	7.38	0.0217	
C^2^	0.0049	1	0.0049	0.1346	0.7214	
D^2^	0.1257	1	0.1257	3.43	0.0936	
Residual	0.3662	10	0.0366			
Cor total	2.76	24

**Table 11:** ANOVA Quadratic model for Flexural strength response.

Source	SS	df	MS	F-value	p-value	
Model	2.50	14	0.1789	7.24	0.0017	Significant
A-OPC	0.4514	1	0.4514	18.26	0.0016	
B-CPA	0.3361	1	0.3361	13.60	0.0042	
C-Fine Agg	0.4585	1	0.4585	18.55	0.0015	
D-Coarse Agg	0.0006	1	0.0006	0.0256	0.8761	
AB	0.0012	1	0.0012	0.0490	0.8292	
AC	0.0151	1	0.0151	0.6099	0.4529	
AD	0.0610	1	0.0610	2.47	0.1472	
BC	0.0047	1	0.0047	0.1902	0.6720	
BD	0.0054	1	0.0054	0.2178	0.6507	
CD	0.1482	1	0.1482	6.00	0.0343	
A^2^	0.2363	1	0.2363	9.56	0.0114	
B^2^	0.3337	1	0.3337	13.50	0.0043	
C^2^	0.0013	1	0.0013	0.0508	0.8262	
D^2^	0.4007	1	0.4007	16.21	0.0024	
Residual	0.2472	10	0.0247			
Cor Total	2.75	24				

### Derived coefficient estimates and model equations

In line with the experimental plan and subsequent statistical fit ANOVA computations, regression analysis enabled the prediction of each response. This analysis was conducted using Design Expert software, exploring the interaction between variables and responses. The CCD experimental design data facilitated the evaluation of mathematical prediction equations, as illustrated in Table 12. The equations, in terms of coded factors, could be employed to make predictions regarding the response for specified levels of each factor. These predictions were formulated as a function of the factors A, B, C, and D, representing the proportion of cement, CPA, fine aggregates, and coarse aggregates, respectively^51^.

**Table 12:** Developed quadratic model equations.

	Intercept	A	B	C	D	AB	AC	AD	BC	BD	CD	A^2^	B^2^	C^2^	D^2^
Sqrt (Compr. Strength)	5.001	**0.191**	**− 0.12**	**− 0.16**	− 0.01	0.029	− 0.01	0.078	− 0.03	0.002	**0.110**	− 0.23	**− 0.32**	0.044	0.22
p-values		**0.002**	**0.024**	**0.005**	0.763	0.553	0.787	0.134	0.519	0.974	**0.044**	0.088	**0.022**	0.721	0.09
Sqrt (Flex. Strength)	2.770	**0.158**	**− 0.14**	**− 0.16**	0.006	− 0.01	− 0.03	0.062	0.017	0.018	**0.096**	**− 0.31**	**− 0.36**	− 0.02	**0.4**
p-values		**0.002**	**0.004**	**0.002**	0.876	0.829	0.453	0.147	0.672	0.651	**0.034**	**0.011**	**0.004**	0.826	**0.002**

p-value shading: p < 0.05 0.05 ≤ p < 0.1 p ≥ 0.1.

Significant values are bold.

### Diagnostics plots

**Figure 7:**
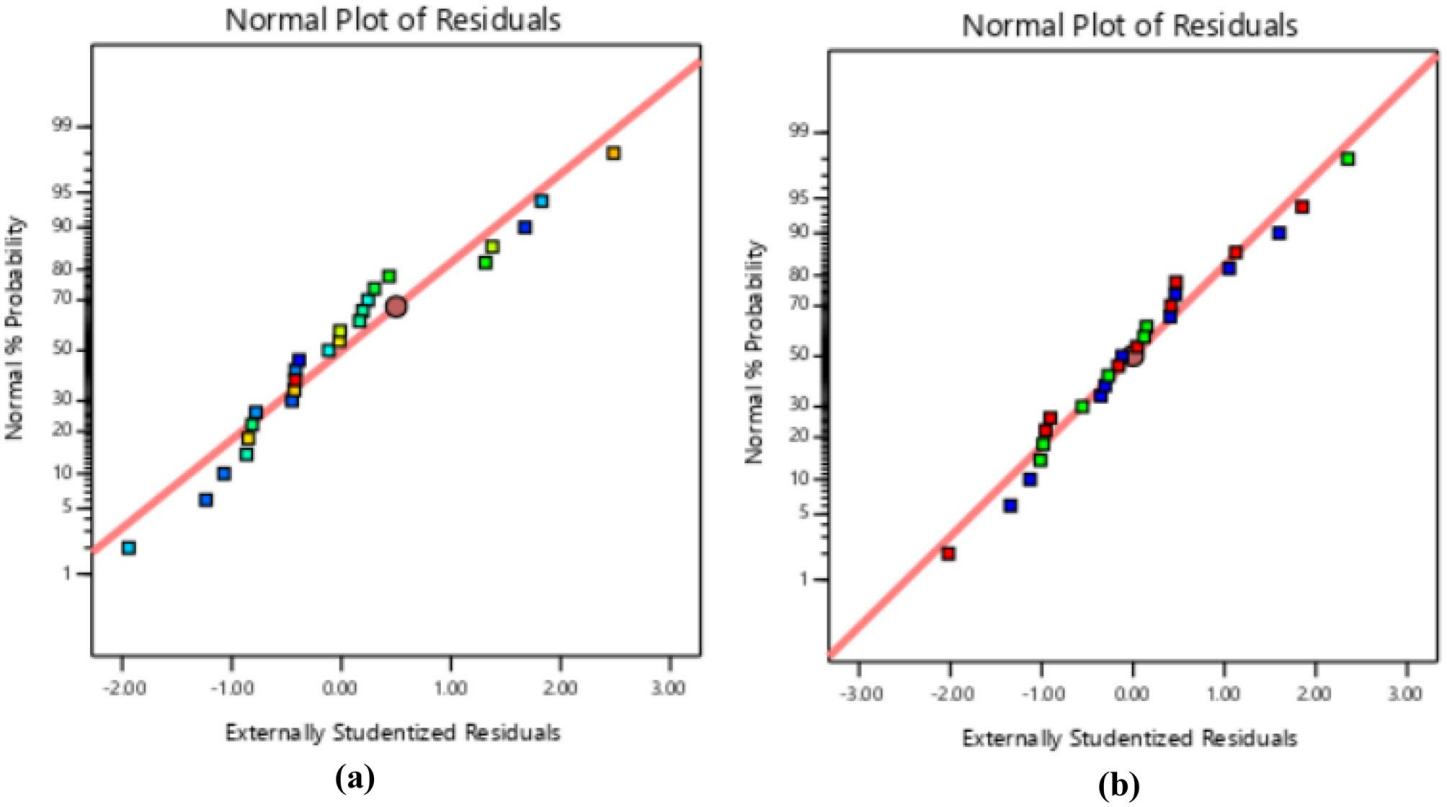
Residuals normal probability plots for the target responses.

**Figure 8:**
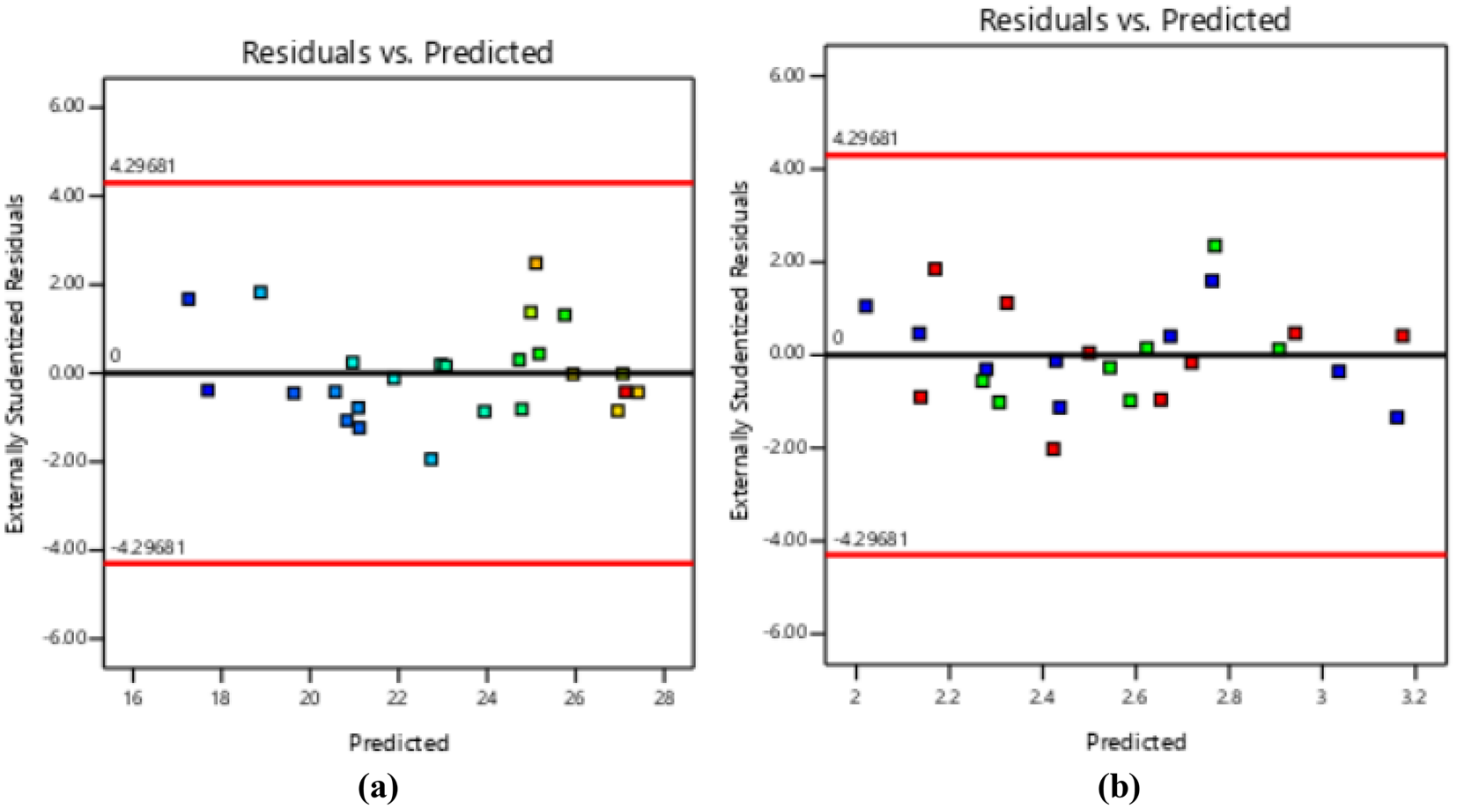
Residuals vs. Predicted plots.

**Figure 9:**
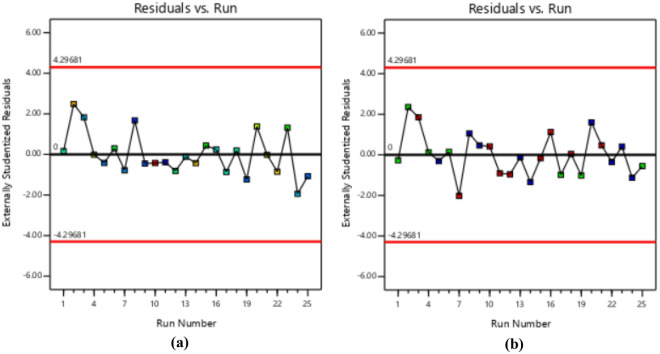
Residuals vs. Experimental Runs plots.

**Figure 10:**
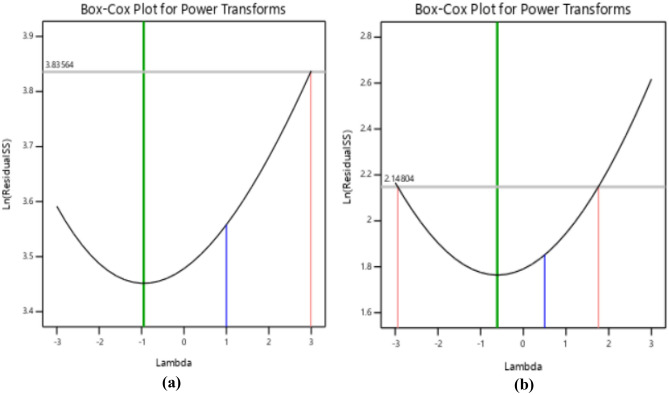
Box-cox plots for power transformation.

**Figure 11:**
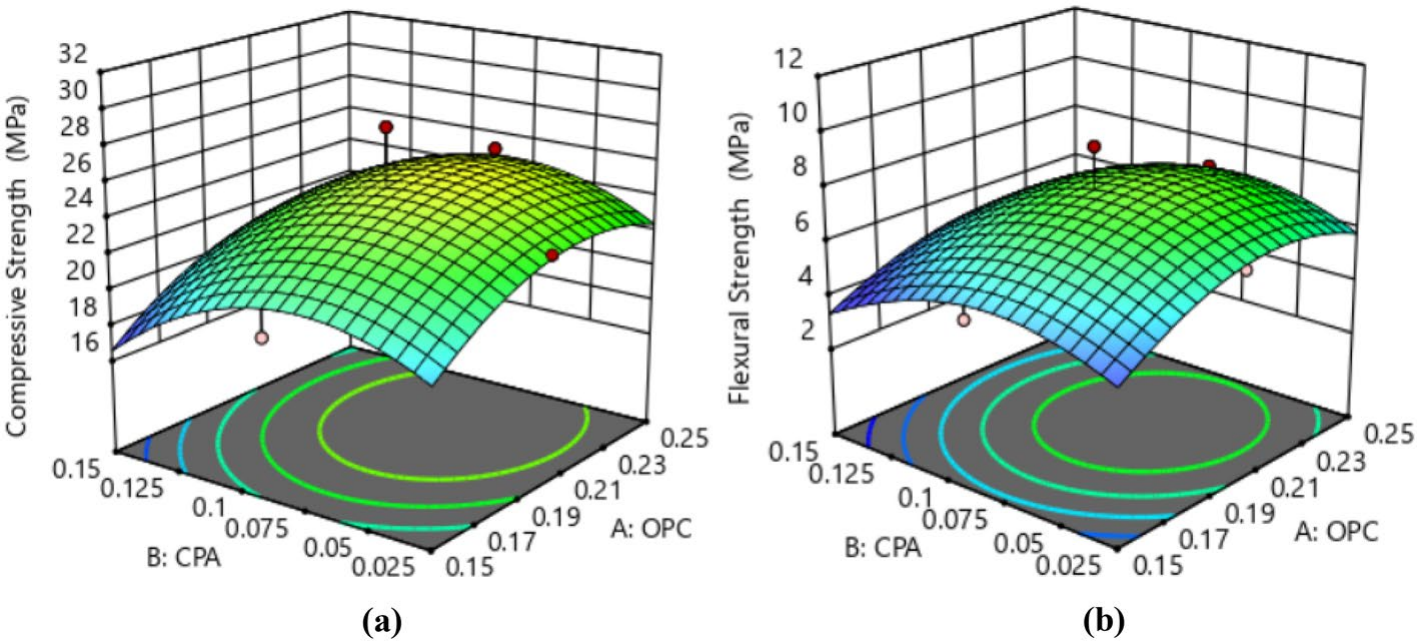
Surface Plot for OPC vs. CPA.

**Figure 12:**
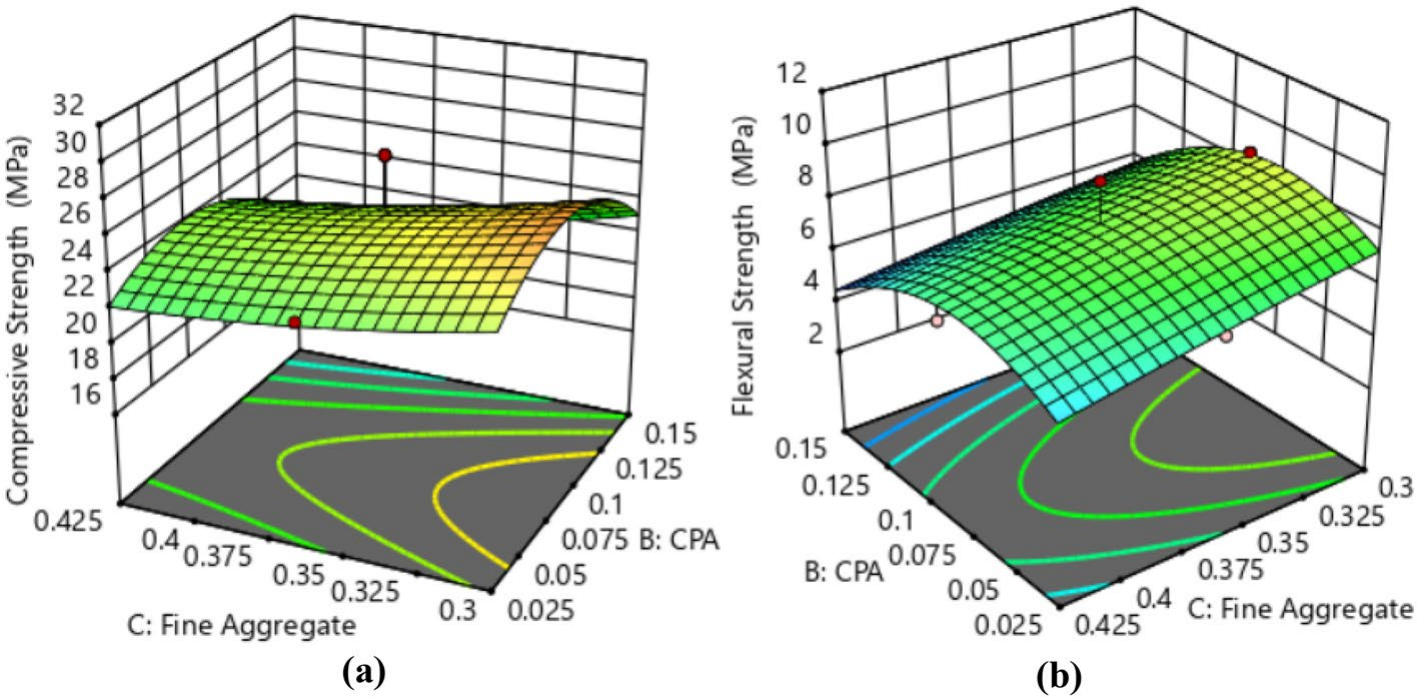
Surface Plot for Fine Aggregate. vs. CPA.

**Figure 13:**
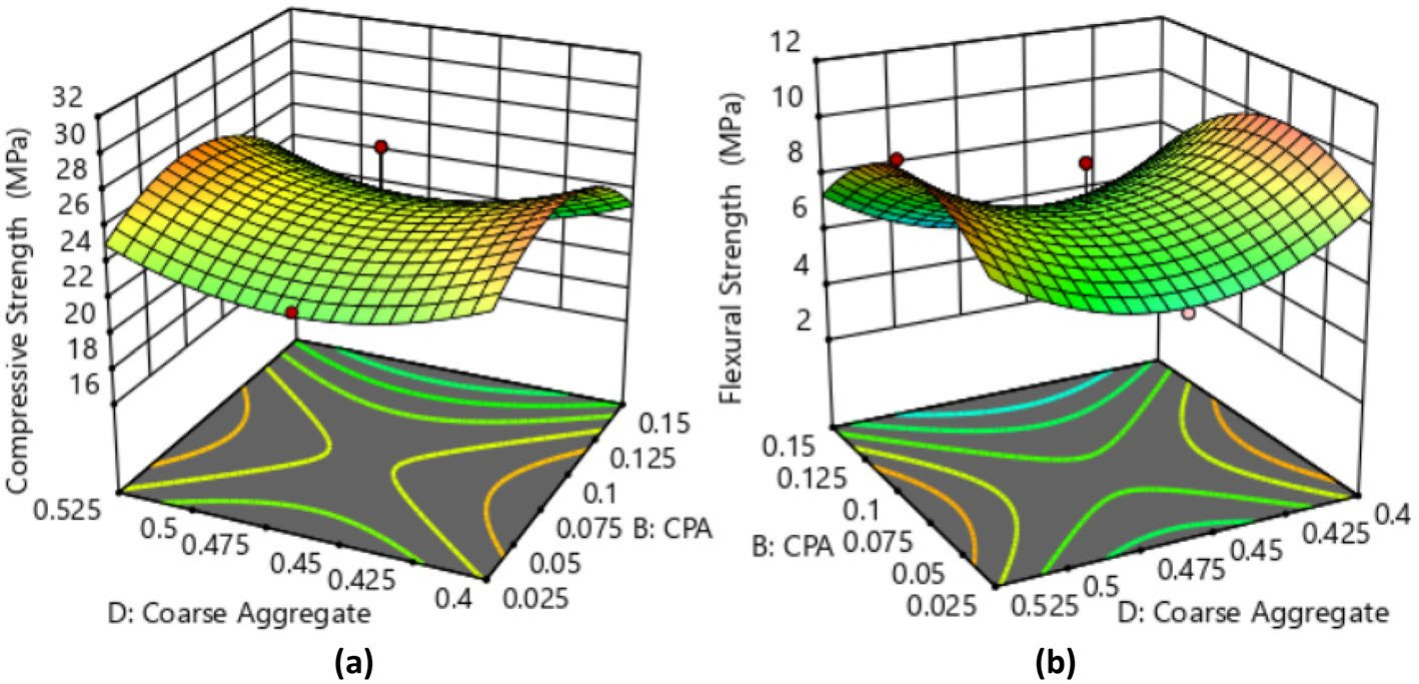
Surface Plot for Coarse Agg. vs. CPA.

The diagnostic statistical graphs, presented as scattered plots of residuals or model prediction errors against the predicted values, serve to assess whether further refinement of the estimation is possible. These graphs are also utilized to gauge the goodness-of-fit of the developed model using studentized residuals, confirming adherence to regression assumption conditions and identifying potential influential observations that could significantly impact the analysis results. It's noteworthy that the standard errors of the derived residuals differ unless the experimental runs' leverages in the design are identical, signifying that raw residuals belong to varying populations and are insufficient for evaluating regression assumptions^52,53^. However, studentized residuals are preferred as they map all normal distributions in different dimensions to a unitary distribution. Regarding the desired response variables, diagnostic statistical tests in this analysis were conducted at upper and lower intervals of ± 4.29681, encompassing predicted vs. residual, normal probability, experimental run vs. residuals, predicted vs. actual, and Box-Cox power transformation. These tests aid in detecting issues with the analysis, including outliers, as depicted in Figs. 7–10. These diagnostic statistical plots provide essential criteria for selecting an appropriate power transformation law to evaluate the effects on the response variables at the current lambda of 0.5. Figures 11–13 illustrate the interaction effect of CPA admixture versus the concrete ingredients concerning the mechanical strength response. The patterns of compressive and flexural strength discernible from these plots aid in comprehending the parameters for optimum responses when CPA is incorporated into the concrete mixture. The results indicate that the addition of CPA led to improvements in the mechanical properties of the concrete, with the best results achieved at an 11.21% replacement of cement with CPA in the mixture^54,55^.

### Optimization analysis

**Figure 14:**
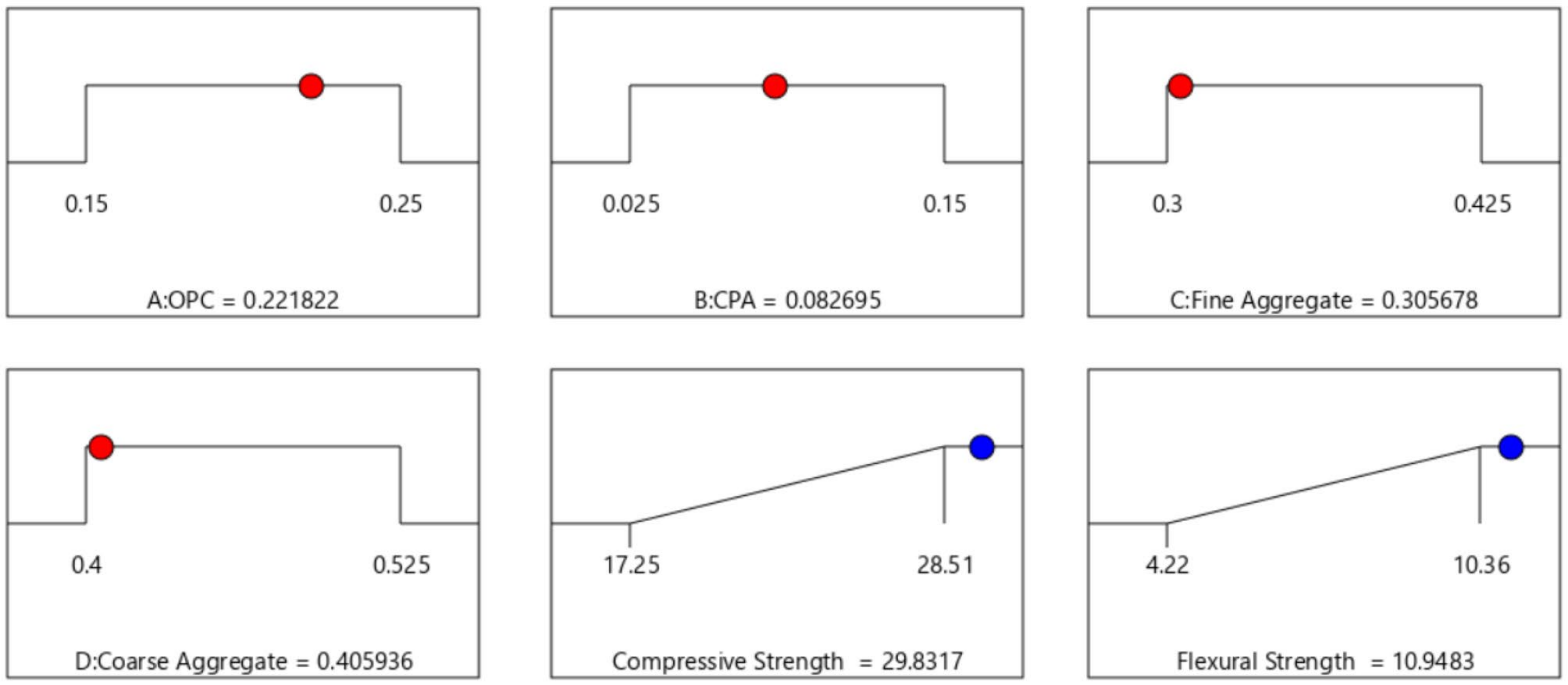
Optimization ramps.

After completing the diagnostic statistical analysis and influence graphical calculations, numerical optimization is undertaken using a desirability function. This function assesses the imposed optimization criteria on the model variables to maximize the target response parameters. To achieve this objective, the characteristics of the objective function are analytically adjusted through modifications to weight functions in accordance with the predetermined model variable criteria^56^. These adjustments consider multicollinearity conditions to enable the attainment of favorable conditions and achieve a desirability score of 1.0 within the boundary conditions of 0 ≤ d(yi) ≤ 1. The optimization component of this experimental design seeks the combination of mixture ratios in the feasible factor space, simultaneously satisfying the formulated and imposed criteria on the response parameters and corresponding factor levels^57^. The primary goal of the optimization is set to maximize the target responses, while the combination ratios of the four components are set within the in-range option to determine the optimal proportion of factor levels that yield a maximum response, as detailed in Table 13. The optimization solution derived from the analytical procedures of the mixture experiment designs is presented in Table 14 and Fig. 14. The obtained results reveal an optimal desirability score of 1.0 at a combination ratio of 0.222:0.083:0.306:0.406, resulting in maximized compressive and flexural strength of 29.832 MPa and 10.948 MPa, respectively^58^.

**Table 13:** Model parameters criteria for optimization.

Name	Goal	lower limit	Upper limit	Lower weight	Upper weight	Importance
A: OPC	Is in range	0.15	0.25	1	1	3
B: CPA	Is in range	0.025	0.15	1	1	3
C: Fine agg	Is in range	0.3	0.425	1	1	3
D: Coarse agg	Is in range	0.4	0.525	1	1	3
Compr. strength	Maximize	17.25	28.51	1	1	3
Flex. strength	Maximize	4.22	10.36	1	1	3

**Table 14:** Optimization solutions.

Number	OPC	CPA	Fine aggregate	Coarse aggregate	Compressive strength	Flexural strength	Desirability	
**1**	**0.222**	**0.083**	**0.306**	**0.406**	**29.832**	**10.948**	**1.000**	**Selected**
2	0.206	0.104	0.330	0.400	28.699	10.447	1.000	
3	0.227	0.072	0.303	0.410	28.901	10.669	1.000	
4	0.215	0.105	0.301	0.407	28.890	10.506	1.000	
5	0.231	0.073	0.316	0.524	28.672	10.505	1.000	
6	0.224	0.074	0.307	0.522	28.757	10.505	1.000	
7	0.202	0.083	0.301	0.402	29.190	10.938	1.000	
8	0.221	0.067	0.304	0.414	29.491	10.406	1.000	

Significant values are in bold.

#### Optimization contour plot

**Figure 15:**
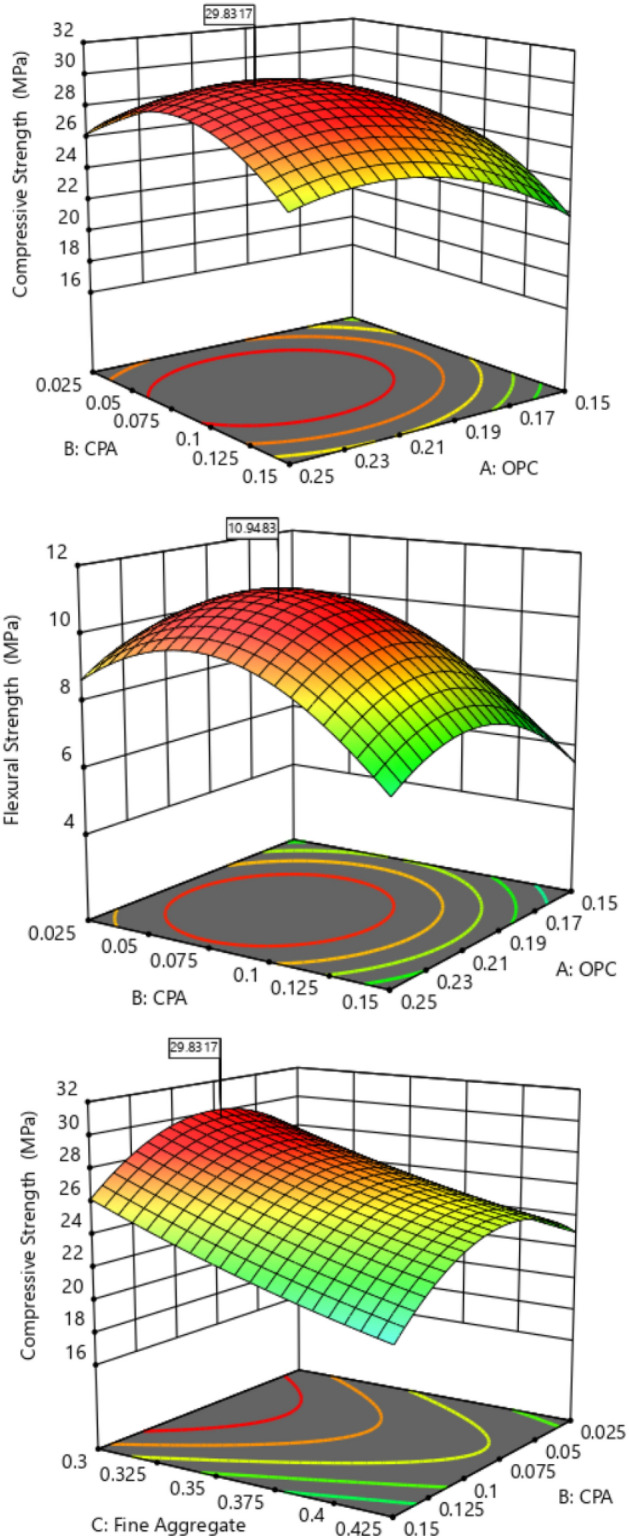

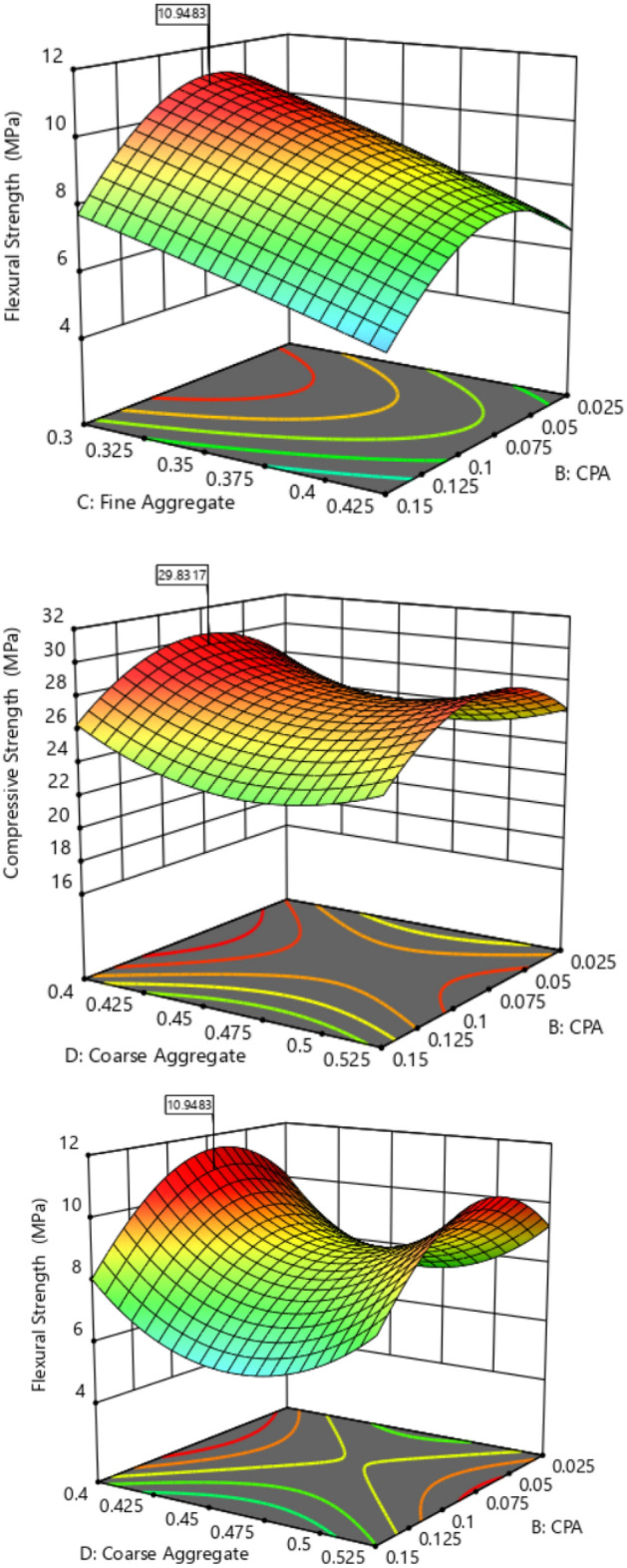

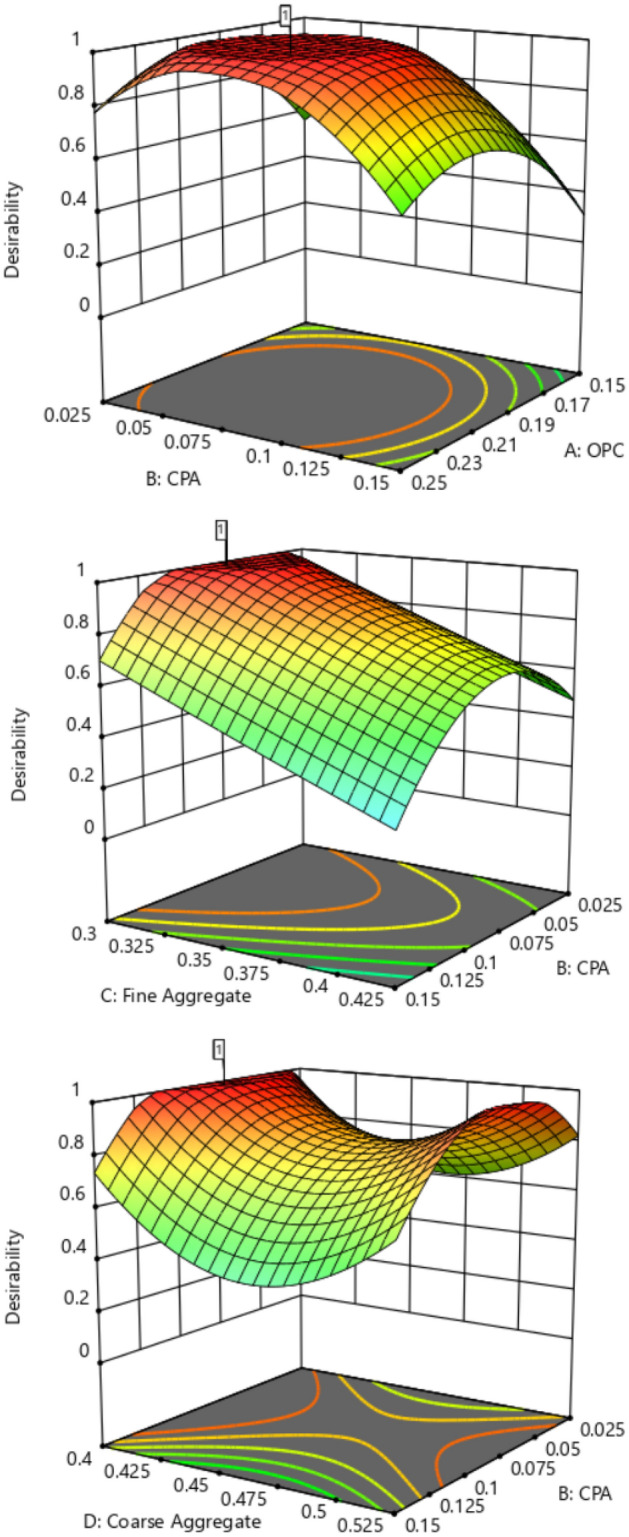
3D Surface Plot for the Optimization Solutions.

The contour plot serves as a crucial tool for visualizing the functional points within the feasible experimental region through iterative mixture design optimization solutions. It is a graphical representation tool for presenting 3D surfaces through contour plotting^59^. Three-dimensional surface plots provide a diagrammatic presentation of the relationships and interactions between the proportions of mixture components and the response parameters^60,61^. The 3D plots for the optimal solution, considering the desirability function and showing the response surface for the corresponding points in the analysis, are depicted in Fig. 15. These graphical solutions illustrate the desirability function of all optimal solutions, adjusted according to the multi-response optimization. From the plot, it is evident that the green surface represents the lowest desirability function, occurring in the range of 0.025–0.05 and 0.15–0.125 fractions of CPA. The highest desirability function is indicated by the red-colored surface, covering the range of 0.075–0.12 fraction of CPA^62–64^.

### Model simulation and validation

**Figure 16:**
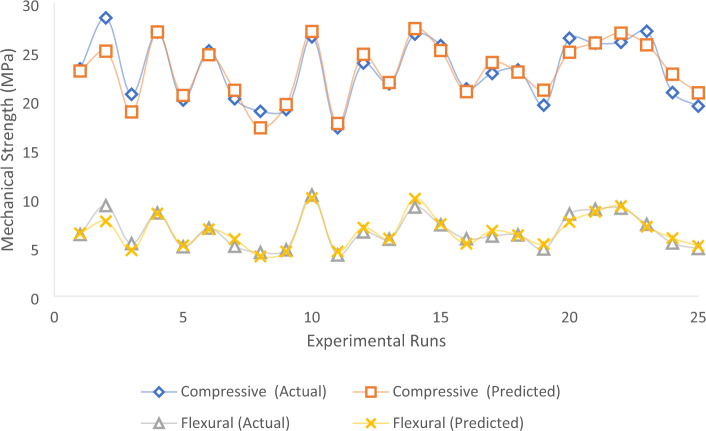
Actual vs. Model Predicted Responses.

This marks the final phase of the model validation process, where we replicate a real-life scenario to provide essential guidance to designers, contractors, and operators regarding the performance of the developed quadratic model^65,66^. The simulation of the model aims to ensure that the validation achieved during statistical diagnostics and inference computations is applicable in real-life situations. Student’s t-test was further employed to determine the statistically significant difference between the simulated model results and the experimental or actual values^64^. A graphical plot illustrating the experimental-derived responses vs. model-simulated results is presented in Fig. 16. The computed results, obtained with the assistance of Microsoft Excel statistical software, are detailed in Table 15. The calculated results reveal p (T ≤ t) two-tail values of 0.9987 and 0.9912 for compressive and flexural strength responses, respectively. The statistical outcomes indicate that there is no significant difference between the actual and model-predicted results, signifying acceptable model performance^67,68^.

**Table15:** T-Test: paired two-sample test for means.

	Compr. actual	Compr. predicted	Flex. actual	Flex. predicted
Mean	23.1052	23.1048	6.66	6.6612
Variance	10.52522	9.064043	3.148717	2.853853
Observations	25	25	25	25
Pearson correlation	0.927893		0.952705	
df	24		24	
t Stat	0.001653		− 0.01113	
P(T < = t) one-tail	0.499347		0.495607	
t Critical one-tail	1.710882		1.710882	
P(T < = t) two-tail	0.998694		0.991215	
t Critical two-tail	2.063899		2.063899	

## Conclusion

The present investigation aimed to optimize the formulation of concrete blended with cassava peel ash (CPA) to achieve superior mechanical properties using a mixture design approach. The study focused on four key parameters: cement content, CPA content, fine aggregate content, and coarse aggregate content, with the primary objectives being to enhance compressive and flexural strength characteristics. Below are the main outcomes derived from the experimental research:

- The research study optimizes a mixture consisting of four components, aiming to evaluate the mechanical strength characteristics of the resulting green concrete. The limits for the design mixture components’ ratios were established based on formulations derived from expert knowledge in relevant literature, ensuring an optimal mixture proportion conducive to maximizing strength response.
- Chemical property analysis affirmed the beneficial pozzolanic characteristics of cassava peel ash (CPA) when utilized as a supplementary cementitious material (SCM). The CPA composition revealed notable percentages of Fe_2_O_3_ (6.02%), Al_2_O_3_ (19.88%), and SiO_2_ (55.93%), summing up to 81.83%. These findings underscore the potential suitability of CPA as an effective SCM in concrete formulations, owing to its significant content of pozzolanic elements.
- The experimental program utilized a face-centered central composite design for laboratory experiments, resulting in a maximum compressive strength of 28.51 MPa and a flexural strength of 10.36 MPa. Subsequently, a quadratic predictive model was developed using the laboratory data, and statistical analyses were conducted to assess the datasets. Through numerical optimization and graphical statistical computations, the optimal levels of mixture ingredients were identified, resulting in a desirability score of 1.0 at a mix ratio of 0.222:0.083:0.306:0.406. This optimal composition led to enhanced compressive and flexural strengths of 29.832 MPa and 10.948 MPa, respectively.
- Adequacy tests performed on the generated model demonstrated a robust correlation between laboratory results and model-simulated values, as confirmed by the student's t-test. These findings underscore the effectiveness of the CCD method in optimizing mixture compositions to achieve desired concrete properties, thereby offering valuable insights for enhancing the mechanical performance of green concrete formulations.

### Recommendation for future research

1. Investigation of Additional Parameters: Future studies could explore the impact of varying parameters such as water-cement ratio, curing conditions, and particle size distribution of cassava peel ash (CPA) on the mechanical properties of concrete. This comprehensive approach would provide a more nuanced understanding of the factors influencing concrete performance.
2. Durability Testing: Given the importance of long-term durability in concrete structures, future research could focus on evaluating the resistance of CPA-blended concrete to environmental factors such as freeze–thaw cycles, sulfate attack, and alkali-silica reaction. Conducting accelerated aging tests and field exposure studies would provide valuable insights into the durability performance of CPA concrete.
3. Sustainability Assessment: Further studies could assess the environmental impact of utilizing cassava peel ash as a supplementary cementitious material in concrete production. Life cycle assessments and carbon footprint analyses could be conducted to quantify the environmental benefits of incorporating CPA and compare them with traditional concrete formulations.
4. Optimization of Mixture Design: Continuation of research into optimizing the mixture design of CPA concrete using advanced statistical methods, such as artificial intelligence algorithms, could further enhance the mechanical properties of concrete while minimizing material usage and costs.
5. Field Applications and Performance Monitoring: Real-world implementation of CPA concrete in construction projects followed by systematic performance monitoring would provide valuable data on its behavior under actual loading and environmental conditions. Long-term monitoring of structures built with CPA concrete would enable the assessment of its structural integrity, durability, and sustainability in practical applications.

## Data Availability

All data generated or analyzed during this study are included in this published article.
